# Current status and global trends in self-care among patients with heart failure: challenges and opportunities

**DOI:** 10.20517/jca.2026.58

**Published:** 2026-08-10

**Authors:** Barbara Riegel, Christopher S. Lee, Tiny Jaarsma, Anna Stromberg

**Affiliations:** 1 Center for Home Care Policy & Research at VNS Health, New York, NY 10017, USA.; 2 Boston College William F. Connell School of Nursing, Chestnut Hill, MA 02467, USA.; 3 Department of Health, Medicine and Caring Sciences, Linkoping University, Linkoping SE-58183, Sweden.; 4 Department of Cardiology, University Medical Center Utrecht, Utrecht 3584 CX, The Netherlands.; 5 Department of Health, Medicine and Caring Sciences and Department of Cardiology, Linkoping University, Linkoping SE-58183, Sweden.

**Keywords:** Heart failure, self-care, self-management, medication adherence, pathophysiology, policy

## Abstract

Self-care effectively improves outcomes in adults with chronic conditions such as heart failure (HF). Yet engaging patients in self-care maintenance, self-care monitoring, and self-care management has proven to be difficult for patients and providers. In this review we define self-care as a decision-making process involving the choice of behaviors that maintain physiologic stability and the response to symptoms when they occur. The three core self-care behaviors of maintenance, monitoring, and management are defined and situated within their theoretical context. Available approaches to measurement are described. Details of what is known about self-care maintenance, self-care monitoring, and self-care management from the global literature are synthesized, focusing on efforts to promote HF self-care behaviors and illustrating the challenges experienced by patients. Barriers to and facilitators of HF self-care are described. Hypothesized and known links between HF self-care and pathophysiology such as myocardial stretch, systemic inflammation, neurohormonal activation, and renal function are reviewed. Guidance is provided on how to design effective self-care interventions. Opportunities for future research are described. The article ends with comments on implications for policy to position self-care as a pillar of universal health coverage.

## Graphical Abstract

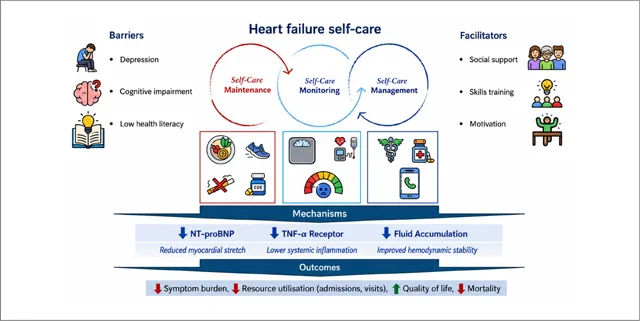

## INTRODUCTION

Self-care has been shown to significantly improve quality of life, symptom burden, and psychological outcomes, and may contribute to reduced healthcare utilization and better survival in patients with heart failure (HF)^[1–3]^ and various other chronic conditions^[4]^. Numerous self-care behaviors such as medication adherence and symptom monitoring are vital for HF^[5]^. Self-care is so important that both the U.S.^[6]^ and European HF guidelines^[7]^ specify self-care education and the need to support self-care among all patients with HF as class I recommendations. Yet many of these behaviors are difficult for older adults due to a complex interplay of factors including cognition^[8]^, psychosocial profile, cultural context, and resource availability^[9]^. In developing countries, self-care is particularly important because such behaviors are often the only resource available.

In the US, beginning in 2027, the Centers for Medicare and Medicaid Services (CMS) will require that some cardiologists participate in a new merit-based Ambulatory Specialty Model payment system for HF. The new program focuses on prevention and upstream management of high-cost chronic diseases such as HF to reduce avoidable hospitalizations. We anticipate that providers will be encouraged to promote self-care behavior. The purpose of this review is to describe our current understanding of HF self-care behaviors, illustrating challenges experienced by patients, and opportunities for intervention and future research.

### Defining characteristics of self-care

**Figure 1.**
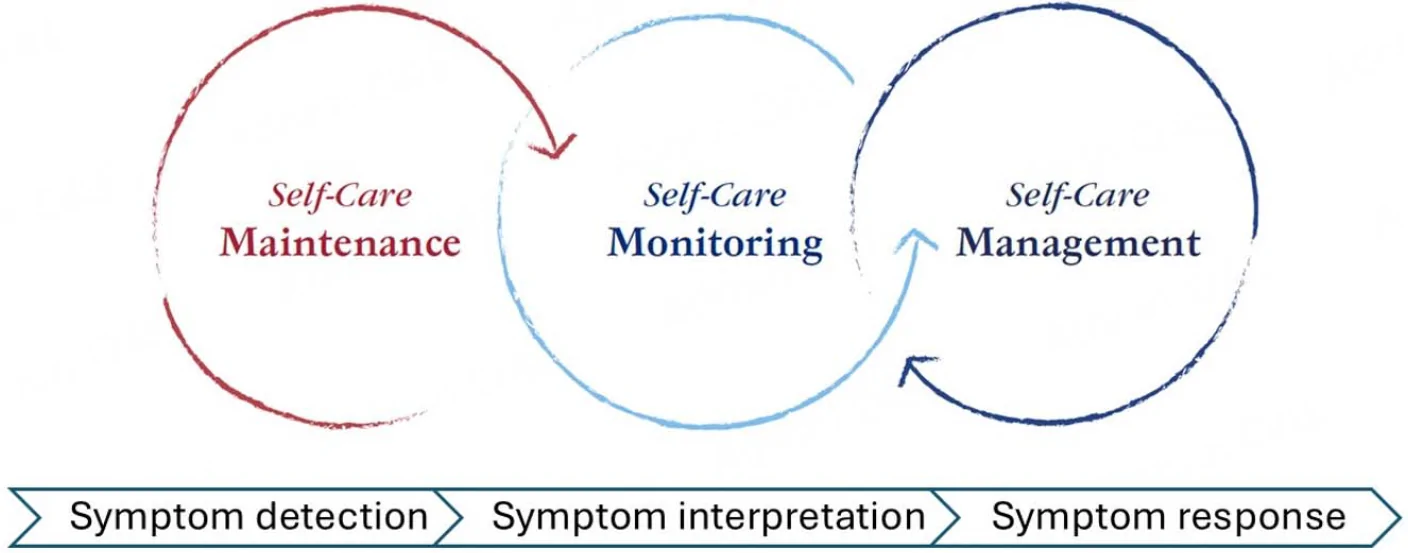
Major concepts in the middle range theory of self-care of chronic illness. Reprinted with permission^[12]^.

We have defined self-care as a process involving decisions about the choice of behaviors that maintain physiologic stability and the response to symptoms when they occur^[10]^. Three core self-care behaviors are maintenance, monitoring, and management [Figure 1]. Self-care maintenance refers to behaviors performed to preserve health, maintain physical and emotional stability, or improve well-being. Many self-care maintenance behaviors mirror the recommendations of healthcare providers (e.g., smoking cessation, healthy eating) or address the medical regimen (e.g., taking medication as prescribed) [Table 1]^[11]^. Self-care monitoring is the process of routine body listening, self-awareness, and vigilant surveillance. Monitoring of one’s health (e.g., energy and body weight) is a common human behavior, but in the setting of a chronic illness such as HF, checking blood pressure, weighing routinely, and monitoring emotions help to achieve physical and emotional stability. Self-care management is the most complex and demanding of the three core behaviors because it involves the response to signs and symptoms when they occur. Individuals who are successful in self-care management can mentally simulate options and decide on a course of action. If a response is needed, self-care management involves seeking advice or using a treatment such as behavioral change or medicine. Decision-making is emphasized because self-care maintenance, self-care monitoring, and self-care management all require that patients make a decision to engage in the behavior. Although the three behavioral domains of self-care are relevant to all patients living with HF, it is important to note that the vast majority of evidence comes from studies focused on HF with reduced ejection fraction or a mix of patients with varying phenotypes.

A common misunderstanding about self-care, promoted by the marketing industry, is that self-care involves bubble baths, massage, and indulgence. We refer to this as self-soothing, which involves activities that provide distraction and comfort in difficult times^[12]^. Self-soothing is useful to temporarily relieve stress and anxiety, but there is currently little evidence that self-soothing improves clinical outcomes in HF.

### Theory and measurement

We have proposed two major theories of self-care; one is specific to HF^[10]^ and one can be used with any variety of chronic illnesses. The HF theory was developed first and used to build the Middle Range Theory of Self-care of Chronic Illness^[12]^. The major difference between the two theories was the addition of the concept of self-care monitoring in the Middle Range Theory of Self-care of Chronic Illness. Recognizing the importance of monitoring, we updated the HF theory to include a HF-specific concept related to self-care monitoring - symptom perception, described below. These two theories have been used to design several different self-report instruments focused on self-care. All the instruments are harmonized with the theories to assess self-care maintenance, self-care monitoring, and self-care management in three separately scored scales. Scores on each scale are standardized 0–100 and a score less than 70 is considered inadequate self-care. The theories and instruments are used by interdisciplinary scientists and clinicians worldwide, which has provided a rich body of data regarding self-care globally.

## WHAT HAVE WE LEARNED?

### Self-care maintenance

The first self-report instrument developed was the European HF Self-Care Behaviour Scale (EHScFBS)^[13]^, followed soon thereafter by the Self-Care of HF Index (SCHFI)^[14]^. Both instruments are made freely available to investigators. The SCHFI is available on our website (www.self-care-measures.com) where 26 different language translations are posted. The EHScFBS is available in 33 languages (https://liu.se/en/research/european-heart-failure-self-care-behaviour-scale/ehfscb-versioner-Versions-LinköpingUniversity). With so many translations, we have learned a great deal about self-care practices globally. A recent systematic review of 39 studies from Asia (Iran, China, Korea, Taiwan, Thailand, and Jordan), North America (USA, Mexico, and Canada), Europe (Italy and UK), Brazil, and Australia (8,958 patients) found that self-care maintenance, overall, was inadequate (mean 58.16 (CI: 54.39–61.94 average standardized score)^[15]^. In the 36 studies reporting an average score for self-care maintenance, scores ranged from 20 in a study from Iran^[16]^ to 76 in a study conducted in the US^[17]^. The average score for self-care maintenance was above 70 (i.e., adequate) in just four of the 36 studies.

In a study of data from 15 countries across Europe, Australasia, North and South America, we found that most patients with HF (5,964 patients) reported taking their medications as prescribed^[18]^. However, despite such reports, medication adherence is actually worse than most providers realize^[19–22]^. In one study, 90% of HF patients self-reported at least intermittent nonadherence^[23]^. When medication adherence was measured objectively, Giordano *et al*.^[19]^ found that 22% of patients with heart disease were nonadherent to their medication regimen. Self-reported adherence may be subject to social desirability bias, whereas objective measures capture only pill-taking behavior without reflecting patients’ decision-making process.

Factors associated with medication nonadherence in patients with HF are multifactorial. Reasons may be regimen-related (e.g., cost of medicines^[21,22,24]^, polypharmacy^[22,25]^, medication complexity, side effects)^[25]^, patient-related (e.g., depression, age, cognitive function, poor sleep, comorbidities)^[25]^, disease-related (e.g., symptom-free periods, health status^[26]^), and/or provider-related (poor communication^[27]^, lack of trust^[28]^, lack of education and support^[29]^, infrequent visits with the provider^[30]^). Notably, missing medications is usually unintentional but in one study, 27% reported intentional nonadherence defined as skipping, reducing the dose, or deliberately discontinuing a medication^[23]^. Medication adherence was described as an act of personal choice. Some respondents perceived the diagnosis of HF as low threat. Others did not believe that taking medication was necessary. These patients described disliking medication taking or that medication side effects influenced their behavior^[23]^.

In the study of patients from 15 countries discussed above, exercise rates were low^[18]^. Specifically, exercise was the self-care behavior least likely to be endorsed by respondents. In HF, this may be due to exercise intolerance caused by reduced cardiac output, impaired oxygen delivery to muscles, frailty, or fatigue^[31]^. Other factors include fear of damaging the heart, shortness of breath, limitations due to physical factors such as edema, and historically outdated advice to rest^[32]^. Many people simply lack interest in exercise or have little motivation to begin or maintain the behavior^[32]^.

Tobacco use is another self-care behavior that is challenging for patients with HF. A surprising proportion of patients with HF (16%) continue to smoke after diagnosis^[33]^. This is despite data demonstrating increased risk of death and readmission, poor health status, ventricular tachycardia, and arterial stiffness in HF patients who smoke^[33]^. Continued smoking may be due to nicotine addiction, the use of cigarettes for stress relief, and habitual routines. Social factors—such as having smokers in the household—may make smoking cessation difficult^[34]^. Another possible reason for continued tobacco use could be mild early HF symptoms. Persons with mild symptoms may not be motivated to quit^[33]^.

Recent data related to dietary sodium and fluid restriction^[35,36]^ have spurred major changes in clinical guidelines for HF^[6]^. Historically, dietary sodium was restricted in persons with HF based on a physiologic rationale that consuming sodium causes fluid retention. Fluid restriction of 1.5 to 2 L/day was advocated in HF, believing that it would alleviate symptoms of fluid overload. However, there is little evidence to support either sodium or fluid restriction^[37,38]^. Further, there is now evidence indicating that excessive, long-term restriction of dietary sodium (typically < 2,300 mg/day or sometimes < 1,500 mg/day) can be harmful^[39]^. Strict fluid restriction causes persistent thirst and significantly decreases quality of life^[40]^. In 2024 the European Society of Cardiology published a clinical consensus statement explaining that dietary sodium and fluid restriction are no longer recommended for the management of HF^[41]^ and clinical practice in the US has followed suit.

The randomized FRESH-UP trial questions the need for fluid restriction among patients with stable, symptomatic chronic HF. Comparing a fluid restriction of 1.5 liters with liberal fluid intake, there were no differences in safety outcomes (including hospitalizations or mortality), calling into question the routine use of fluid restriction in this population^[42]^.

### Self-care monitoring

Authors of a systematic review and concept analysis defined self-care monitoring as an activity requiring one to pay attention, be confident, and use routines for tracking symptoms, signs, and actions^[43]^. The performance of effective self-care monitoring requires knowledge, a wish for independence and control, and a commitment to performing the behavior. Doing so may feel burdensome to some patients. Monitoring may also increase their interaction with the healthcare system when changes are perceived^[43]^. However, monitoring can enhance well-being with feelings of independence and confidence^[44]^. In HF, self-care monitoring empowers patients to detect early, subtle changes in signs such as weight gain or swelling and symptoms like fatigue, allowing for proactive, timely interventions that prevent severe acute exacerbations.

The process of recognizing a physical sensation as a symptom is seminal; a bodily sensation must be detected, presumably through body listening. Detection precedes perception, which involves becoming consciously aware of a symptom^[45]^. Once detected and perceived, interpretation and the assignment of meaning occurs, which results in symptom recognition. We demonstrated in a secondary analysis of cross-sectional data from 1,629 adults that symptom recognition mediated the relation between self-care monitoring and autonomous self-care management behaviors^[46]^. Yet, the recognition of HF symptoms is often impaired, probably due to impaired interoception or the perception of internal signals from the body^[47]^. Impaired interoception in patients with HF may be related to defects in the insular cortex that occur in HF^[47,48]^. Other causes of poor symptom perception are living alone and aging^[49]^. In persons with HF, misconceptions about symptoms and a lack of experience with HF symptoms (e.g., New York Heart Association functional class I) may cause problems attributing symptoms to HF^[50]^. Misconceptions may involve issues with integration of information derived from interoceptive sources with cognitive-affective processes such as attention, expectations, memory, and beliefs^[51]^.

### Self-care management

Self-care management is the most challenging of the three core behaviors, especially for patients with HF. Reasons include the high cognitive, emotional, and physical demands of managing complex and variable symptoms^[52]^. Early HF symptoms can be subtle, so they are often hard to detect or are wrongly attributed to aging^[53]^. When patients have multiple diseases, symptoms may overlap, making it difficult to determine how to manage symptoms^[54]^. Cognitive impairment is common in HF, making decisions difficult when symptoms arise. Finally, depression and anxiety are found in many persons with HF, reducing motivation and energy needed to manage symptoms^[5,10]^.

In the systematic review of studies conducted across the world, cited above^[15]^, self-care management overall was inadequate (53.11 (CI: 49.17–57.05) average score). Thirty-five studies reported an average score for self-care management, with standardized scores ranging from 14 in Iran^[16]^ to 68 in the US^[55]^; scores in every country were below the 70 cut point for adequacy.

## BARRIERS TO AND FACILITATORS OF HEART FAILURE SELF-CARE

### Quantitative evidence

Major barriers and facilitators of HF self-care with the highest level of evidence in accordance with American Heart Association methodology^[56]^ are presented in Table 2. In a systematic review and meta-analysis of 65 studies on psychological determinants of HF self-care, depression was associated significantly with worse self-care across studies^[57]^. A recent study confirmed that depressive symptoms negatively influence HF self-care^[58]^.

In a systematic review of 10 studies on cognitive function and HF self-care, the vast majority of studies reported mild cognitive impairment as a significant barrier to HF self-care^[59]^. In the most recent meta-analysis of 14 studies on the same topic, all but one study linked cognitive dysfunction to poor HF self-care^[60]^.

Health literacy is a barrier to HF self-care^[61]^ and critical health illiteracy (i.e., the inability to evaluate critical information) is an independent predictor of HF self-care^[62]^. Health literacy directly shapes patients’ abilities to access, understand, appraise, and apply health information in daily decision-making^[63]^. Limited health literacy contributes to poorer outcomes through pathways involving reduced disease knowledge, impaired self-efficacy, and suboptimal self-care behaviors^[64]^. In HF, this is particularly evident in symptom perception and response that are essential for timely self-care. Patients with low health literacy are at increased risk of adverse outcomes such as hospitalization and mortality^[65,66]^. Importantly, accumulating evidence demonstrates that health literacy is modifiable through targeted interventions^[67]^. More broadly, systematic reviews indicate that digital interventions can improve health literacy and related self-care skills across chronic disease populations^[68]^.

Support from others is increasingly recognized as a key facilitator to HF self-care. In a recent systematic review of 11 studies on social support and self-care in HF and coronary artery disease, social support was positively associated with HF self-care across studies^[69]^.

### Qualitative evidence

Nearly 25 years ago, we presented known barriers and facilitators of HF self-care in the words of those living with the disorder^[70]^. At that time, key barriers to effective HF self-care included physical limitations, coping with HF treatments, lack of knowledge and misconceptions about HF, negative emotions, multiple comorbidities, and personal struggles. In 2013, a meta-synthesis of qualitative studies revealed that most of these barriers to effective HF self-care persisted^[71]^. In addition, several barriers related to the care environment were identified as barriers to HF self-care including conflicting values between patients with HF and the healthcare team, dissatisfaction with care, and limited communication about self-care from clinical providers. Most recently, poor communication and collaboration were identified as key barriers to effective HF self-care from the perspective of patients with HF, their families, and their clinical providers^[72]^.

## LINKS BETWEEN HEART FAILURE SELF-CARE AND PATHOPHYSIOLOGY

**Figure 2.**
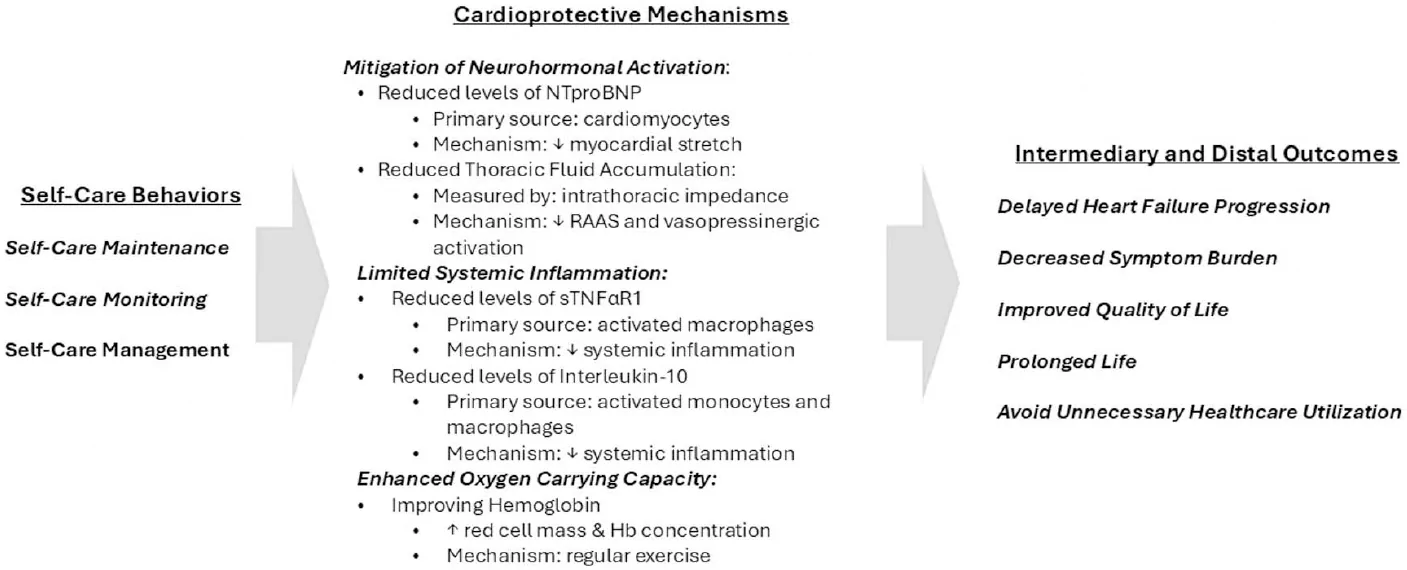
Established clinical associations between pathophysiological biomarkers and heart failure self-care. Based on empirical evidence, effective self-care behaviors are thought to be cardioprotective and therefore improve outcomes in persons with heart failure. There is evidence that effective self-care is associated with mitigated neurohormonal activation as evidenced by lower levels of myocardial stretch and thoracic fluid accumulation. There is additional evidence from multiple sources that effective self-care behaviors are associated with limited systemic inflammation based on multiple pro-inflammatory biomarkers. Finally, there is evidence that effective self-care behaviors, exercise in particular, are associated with erythropoiesis that partially offsets anemia of chronic illness. Hb: Hemoglobin; NTproBNP: amino-terminal B-type natriuretic peptide; RAAS: renin-angiotensin-aldosterone system; sTNFαR1: soluble receptor for tumor necrosis factor alpha type 1.

In 2009, we published hypothetical mechanisms through which effective HF self-care influences outcomes, including neurohormonal deactivation and limited inflammation^[73]^. Presently, many of the proposed mechanisms remain hypothetical, such as avoidance of extreme escalations of pharmacotherapeutic agents and limited myocardial hibernation. But there are established clinical associations between biomarkers of specific aspects of HF pathophysiology and effective HF self-care [Figure 2]. First, there is evidence that better self-care is associated with lower levels of biomarkers of myocardial stretch and systemic inflammation in HF^[74]^. Specifically, better self-care management behaviors are associated with lower levels of N-terminal pro-B-type natriuretic peptide, which is secreted by cardiomyocytes in response to stretch and has high diagnostic^[75]^ and prognostic validity^[76]^. Better self-care management is also associated with lower soluble tumor necrosis factor α receptor type 1 that is elevated as part of the pro-inflammatory phase of the response to tissue injury in HF^[77]^ and has significant prognostic validity^[78]^. As such, effective self-care may be helpful in reducing myocardial stretch and systemic inflammation. More recently, others have shown that better self-care behaviors in general may be associated with worse biomarkers of renal function (i.e., glomerular filtration rate of creatinine as a clinically relevant metric of end-organ dysfunction)^[79]^. This counter-intuitive finding may be due to patients with worse end-organ damage related to HF being driven to engage more in self-care as the signs and symptoms become more burdensome. In that same study, the specific self-care behavior of regular exercise was associated with better renal function and higher hemoglobin production (anemia of chronic illness is common in HF), and lower biomarkers of systemic inflammation^[79]^. Of particular note, certain self-care behaviors that are no longer recommended in guidelines such as restricting fluid and sodium intake were associated with worsening renal function and worse hemoglobin production, respectively^[79]^.

Second, there is evidence that better HF self-care is associated with fewer and less severe episodes of fluid accumulation measured by intrathoracic impedance^[80]^. These subclinical accumulations of lung water related to neurohormonal activation and fluid and sodium retention are important clinically because they often precede unplanned healthcare utilization in HF^[81]^. In more recent studies, lung impedance is being integrated into HF self-care monitoring strategies^[82]^.

## DESIGNING EFFECTIVE SELF-CARE INTERVENTIONS

**Figure 3.**
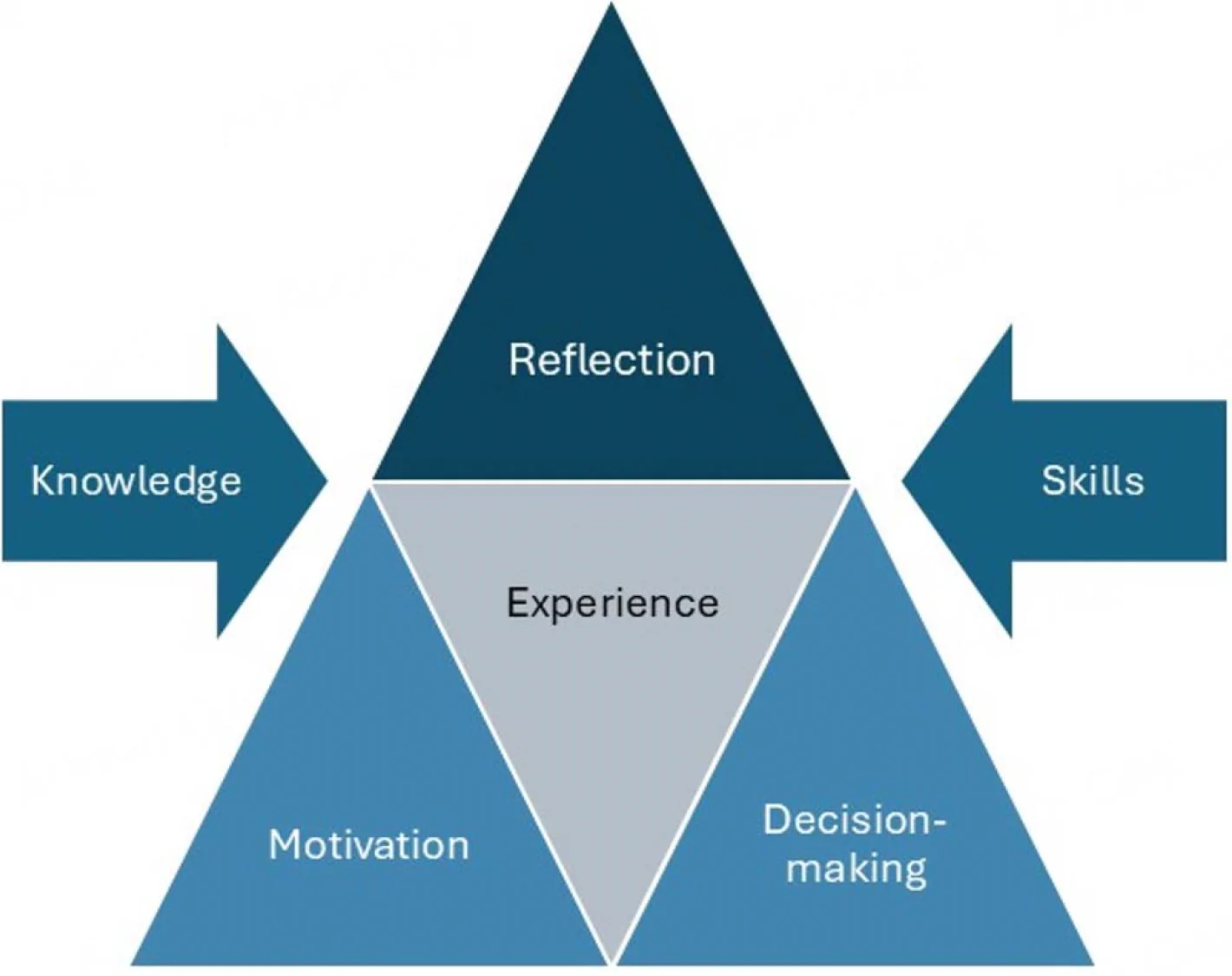
Essential intrapersonal requirements for effective self-care. Some level of expertise in these six elements is required for effective self-care. Reprinted with permission^[12]^.

In designing interventions, six intrapersonal factors have been identified as essential for self-care [Figure 3]^[12]^. These requirements - experience, knowledge, skills, reflection, decision-making, and motivation - can be used in the design of self-care interventions. We refer to these six factors as requirements because even the best intervention will fail if the patient does not know what to do or is not motivated to perform the self-care behavior [Table 3].

Self-care involves both knowledge and skills, but too often providers focus on knowledge without addressing skills. For example, patients with HF may be taught the importance of taking their medications regularly but rarely are skills such as how to manage diuretics during dehydration due to fever or stomach flu or how to adapt physical activity during extreme weather addressed. Effective strategies for teaching skills include breaking information into a few manageable, key points, involving caregivers, and using visual aids. Teach-Back - asking patients to explain what they were told in their own words - is advocated when discussing complex information^[83]^. Such an approach stimulates reflection for both the provider assessing comprehension and for the patient processing information. It prompts the provider to reflect on their communication effectiveness (e.g., “Did I explain this clearly?”) and encourages patients to actively process and verbalize their understanding^[84]^.

As noted above, symptom perception is poor in patients who lack experience with symptoms^[85]^. That is, patients who are rarely symptomatic lack an opportunity to practice making decisions about symptoms. Rather than waiting for them to experience symptoms, potential scenarios can be simulated to enhance their understanding of warning signs^[86]^. Hypothetical situations can be used to build decision-making capability. Coach patients in describing their symptoms and choosing treatment options^[87]^. Examples of other successful approaches to supporting patients in making better self-care decisions are discussed in a systematic review of 58 studies of self-care support strategies in primary care settings^[88]^.

We learned in a recent meta-analysis of self-care interventions in patients with various chronic conditions that comprehensive self-care interventions (i.e., those addressing self-care maintenance, self-care monitorinĝ and self-care management) were most effective in improving outcomes^[89]^. This evidence leads us to encourage investigators and providers to address all three self-care behaviors - self-care maintenance, monitoring, and management - when caring for patients with HF. Only encouraging them to take their medicines as prescribed will not be nearly as effective as talking with them about monitoring symptoms and what to do about symptoms when they occur in addition to preventing symptoms with medication adherence.

Self-care is not a static behavior^[90]^. Some patients have persistently good self-care, some have persistently poor self-care, others improve or deteriorate over time. Declining or persistently poor self-care is associated with higher hospitalization rates and worse outcomes, highlighting the need for sustained, tailored self-care support.

In a scoping review of characteristics of self-care interventions in chronic conditions including HF, it was revealed that most self-care interventions employ face-to-face group or individual sessions focused on education, advice, and/or instruction as opposed to other modes and types of delivery. Moreover, the most common behavioral change technique other than self-monitoring of behavior used by more than one third of interventions was goal setting and problem solving^[91]^. In our recent meta-analysis of 140 self-care trials in chronic illness including HF, we provided new evidence that interventions that use face-to-face *vs*. other modes of delivery, and those that provide feedback on behaviors, include social support, and use reminders *vs*. other behavioral change techniques are more efficacious in improving clinical outcomes^[89]^. As such, the mode of delivery and specific behavioral change techniques being tested in future trials should be selected based on the best available evidence.

Even when seemingly appropriate self-care interventions are developed, unmitigated risk of bias hampers our ability to move the science of self-care forward into clinical practice. In both our meta-analysis of self-care interventions and comprehensive self-care interventions, most trials were rated as having a high risk of bias, particularly in the domains of performance and detection bias^[89,92]^. As such, the pace of our ability to develop self-care interventions has not been matched with the level of sophistication necessary to provide robust evidence of intervention efficacy in trials.

## FUTURE RESEARCH

There are several areas that should be of the highest priority in HF self-care research moving forward. First, since self-care monitoring was introduced later in the development of self-care theory and measurement, future research should focus on understanding this behavioral domain in HF, and how it intersects with other behavioral research topics such as interoception^[93,94]^. Second, future research should focus on the intersection between self-care and frailty, a common and co-occurring syndrome in HF^[95,96]^. Third, we need more evidence on the influence of self-care on underlying pathophysiological mechanisms in HF beyond what we have proposed and the limited empirical evidence available. Fourth, we have limited information on the role of social determinants of health in HF self-care^[97]^, despite their well-established importance in other aspects of HF care^[98]^. Fifth, more inquiry is needed into why communication between clinical providers and patients with HF is so poor and inadequate when it comes to information about self-care. Sixth, we need more evidence on effective strategies to improve HF self-care in the context of known barriers such as depression^[99]^. Seventh, we need more insights into HF self-care in the context of relationships and families more broadly given the importance of care partners and caregivers in supporting self-care^[100]^.

Finally, we need more study of digital health technologies such as wearables, smartphone-based symptom tracking, implantable sensors, and emerging artificial intelligence (AI)-supported decision tools. These approaches are increasingly reshaping HF self-care by enabling continuous monitoring, early detection of clinical deterioration, and personalized support for decision-making. Interventions using digital platforms such as telemonitoring systems, mobile health (mHealth) applications, and remote patient monitoring have demonstrated improvements in self-care behaviors, disease-specific knowledge, and clinical outcomes such as mortality and hospital readmissions while supporting patient engagement in self-care^[101,102]^. These approaches represent a critical extension of traditional self-care monitoring models and are likely to play a central role in future HF research, care pathways, and population health strategies.

## CLINICAL IMPLICATIONS

There is robust evidence supporting the effectiveness of self-care interventions on both patient-reported and clinical outcomes, which supports their use in real-world clinical settings. With the support of international guidelines recommending structured support for behaviors such as symptom monitoring, medication adherence, and timely response to symptoms, many healthcare systems have adopted multidisciplinary models of care to support such approaches^[5]^.

Despite this strong evidence base and guideline endorsement, the implementation of self-care support in routine clinical practice remains suboptimal. Clinicians often face substantial practical barriers, including misaligned reimbursement structures that prioritize acute care over longitudinal support, workforce shortages and increasing demands on specialized HF services, fragmented transitions between care settings, and limited consultation time during clinic visits. These factors constrain opportunities to provide comprehensive, person-centered self-care support and limit the translation of evidence into practice.

In this context, emerging digital solutions, including AI, may offer promising opportunities to bridge the gap between the needs of patients and the capacity of healthcare systems. AI-enabled tools can support early detection of clinical deterioration, and real-time remote monitoring, enabling more proactive and personalized care delivery to support self-care. Furthermore, AI-driven decision support systems and digital platforms may enhance patient self-care by facilitating symptom monitoring, providing tailored feedback, and supporting behavior change outside traditional clinical encounters. Nevertheless, successful integration of AI into HF clinical care requires careful consideration of implementation challenges, including data quality, system integration, clinician acceptance, and ethical considerations. Addressing these barriers will be essential to realize the full potential of AI in augmenting self-care support and improving outcomes for patients with HF. At this time most AI applications for self-care are still in the very early developmental stages^[103]^, far from being able to be implemented in clinical practice. Further, these approaches are far from being accessible in developing countries.

## CONCLUSION

We have argued that the future of self-care is in intervention research^[104]^. The World Health Organization describes self-care interventions as “promising and exciting” approaches to improving health and well-being because they support the goal of universal health^[105]^. In areas of the US and globally, under-resourced health systems and economic differences influence self-care behaviors by limiting access to resources, education, and medicine^[106]^. Developed countries such as the US typically emphasize individual responsibility, whereas developing countries may rely more on family-based support. Individualistic and collectivistic cultural beliefs interact with other cultural values such as power differentials to shape self-care behaviors^[107]^. Cultures with large power differentials tend to obey those perceived as being a higher authority, such as the physician who prescribes a medicine. Yet, as detailed above, studies show high variance in daily practices such as exercise, tobacco use, and medication adherence, with generally poor adherence worldwide^[15,18]^.

A core principle of universal health coverage is that health is a fundamental human right. Self-care promotes health, yet self-care behaviors vary significantly across countries, largely influenced by cultural, economic, and health system differences. The WHO promotes universal health coverage to ensure all people can access quality health services—prevention, treatment, and rehabilitation—without facing extreme financial hardship or poverty. If self-care is not formally integrated into global and national policies, the long-term consequence is a health system that is unsustainable.

## Figures and Tables

**Table 1.** Examples of self-care behaviors^[11]^

Self-care dimension	Definition	Examples

Self-care maintenance	Behaviors performed to improve well-being, preserve health, or maintain physical and emotional stability	• Try to avoid getting sick (e.g., keep vaccinations up to date) • Reduce time sitting • Get enough sleep • Take prescribed medicines as advised • Maintain good mental health (e.g., mindfulness, yoga)
Self-care monitoring	The process of routine vigilant surveillance, self-awareness, and body monitoring or body listening	• Monitor for changes in weight, swelling, and symptoms, especially during activity • Track mood and stress levels • Look for medication side effects
Self-care management	The response to signs and symptoms when they occur	• Change what you eat or drink • Take an additional medication • Call healthcare provider for guidance • Limit activity until symptoms resolve

Note: This list is not exhaustive.

**Table 2.** Major barriers and facilitators to heart failure self-care

Factor	Mechanisms	Level of evidence (American Heart Association Methodology)^[56]^

Depression^[57,58]^	Lack of motivation Blunting of ability to recognize changes Slower responses to symptoms when they occur	Meta-analysis of non-randomized studies (Level B)
Mild cognitive dysfunction^[59,60,108]^	Limited decision-making capacity Blunting of ability to recognize changes Slower responses to symptoms when they occur	Systematic reviews of non-experimental studies (Level B)
Health literacy^[61,62,109]^	Knowledge enhancement Misconception correction	Narrative reviews of non-experiment studies (Level B)
Social support^[69]^	Instrumental support Confidence building	Systematic reviews of non-experimental studies (Level B)

**Table 3.** Barriers, strategies, and outcomes associated with the six requirements for effective self-care

Requirements for effective self-care	Key barriers	Recommended strategies*	Measurable outcomes

Experience - provides experiential familiarity, but time alone is not sufficient to build practical skills, self-efficacy, and reflective habits^[12,59,110,111]^	Patients newly diagnosed with HF do not have experience on which to rely. Learning self-care is analogous to learning any new skill	We recommend early counseling of newly diagnosed patients so that effective self-care habits are formed (Level C)	Experience may help to improve self-care
Knowledge - is empowering^[110]^. Health literacy and education provide the information needed to enable self-care^[112–114]^	Patients who do not know what self-care behaviors are needed cannot be expected to perform self-care	Targeted education yields measurable improvements in knowledge about self-care (Level B)^[113]^. Teach-Back is useful when discussing complex information^[83]^	Higher knowledge improves self-care behaviors, treatment adherence, clinical outcomes, and quality of life^[115]^
Skills - self-care success relies heavily on actionable, practiced skills such as dosing medications, adjusting diuretics, interpreting daily weights, reading food labels, tracking subtle physiological changes, and assessing the importance of symptoms^[116,117]^	Skills can be taught but education typically addresses knowledge rather than skill^[118]^. That is, skills are not commonly addressed during brief educational sessions in the hospital and clinic	Learning skills takes time, practice, and behavioral reinforcement (Level B)	Skill in HF self-care can reduce symptom burden, unscheduled care, hospital admissions, and mortality rates. Self-care skill can enhance overall health-related quality of life^[119]^
Reflection - on actions taken during a HF event such as a symptomatic exacerbation can lead to insights and future changes in HF self-care^[120]^	Behavior - including self-care behavior - is extremely difficult to change. Reflection is promoted through discussion with others, so those without access to others may not engage in reflection	Purposeful, guided reflection may allow the person to better understand an illness experience^[120,121]^ Motivational interviewing can improve reflection about specific self-care behaviors^[122]^. (Level A)	Reflection with an active listener can stimulate reflection, change talk, and self-care behavior change^[120]^
Decision-making - poor, delayed, or impulsive decisions regarding daily routines and symptom changes directly accelerate disease progression, while effective decision-making improves HF outcomes	The quality of self-care decisions depends on the patient’s mental state and cognitive functioning^[123,124]^. Impulsivity and perceived stress are negatively associated with self-care behavior while confidence improves decision-making^[125]^	Structured education, cognitive-behavioral strategies, and remote monitoring help patients to make better decisions^[126]^. Shared decision-making can engage caregivers and clinical teams to overcome emotional hurdles and make better decisions^[127]^. (Level B)	Effective decision-making can improve physiological stability and decrease acute hospitalization and cost^[111]^
Motivation - is critical to bridge the gap between knowing what self-care behaviors are needed and actually performing them^[128]^	Low self-efficacy, emotional distress, time constraints, and lack of perceived necessity are core barriers to motivation to perform self-care^[129,130]^	Motivational interviewing can improve HF self-care through its effect on (1) reflection and reframing, (2) genuine empathy, affirmation, and humor, and (3) individualized problem solving (Level B)^[131]^	Motivation can improve self-care behavior, which has been shown to improve clinical outcomes and health-related quality of life

* Level of evidence using the American Heart Association Methodology: Levels indicate the reliability and strength of research. Level A is the most trustworthy and least biased high-quality evidence from multiple randomized controlled trials (RCTs) or meta-analyses. Level B indicates moderate-quality evidence from RCTs or nonrandomized/observational studies. Level C relies on limited data from studies with major limitations or expert opinion/clinical experience^[56]^.

## Data Availability

Not applicable.
